# Gastric Glomus Tumor Presenting With Gastrointestinal Bleed and Pulmonary Embolism: A Rare Entity With Management Dilemma

**DOI:** 10.7759/cureus.25632

**Published:** 2022-06-03

**Authors:** Kosisochukwu J Ezeh, Yasir Rajwana, Bidhan Paudel, Tingliang Shen, Youssef Botros

**Affiliations:** 1 Internal Medicine, Jersey City Medical Center, Jersey City, USA; 2 Pathology, Rutgers New Jersey Medical School, Newark, USA; 3 Gastroenterology and Hepatology, Jersey City Medical Center, Jersey City, USA

**Keywords:** mesenchymal tumors, subepithelial lesions, gastrectomy, ivc filter, anticoagulation, gi bleed, gastrointestinal stromal tumor (gist), gastric glomus tumor, ggt

## Abstract

Glomus tumors are rare neoplasms originating from smooth muscle cells of the glomus body. They rarely involve the gastrointestinal tract, and when they do, they present as acute gastrointestinal bleeds with symptoms such as hematemesis or melena. We present a rare case of a gastric glomus tumor in a 50- year- old male presenting with shortness of breath and gastrointestinal bleed requiring transfusions. Coincidently, he was also found to have a pulmonary embolism that usually would require anticoagulation, which was contraindicated in an active gastrointestinal bleed. He eventually required an inferior vena cava (IVC) filter and underwent a partial gastrectomy. Due to gastric glomus tumor being a rare entity, there is a paucity of data to have a classification and grading or staging system, and tumors are usually considered benign. The exact diagnosis is dependent on histopathological findings as it can mimic a gastrointestinal stromal tumor (GIST). Pulmonary embolism, a common phenomenon, can often be seen in patients with malignancy. Our patient was diagnosed with a glomus tumor which is usually benign. As per our literature search, there are no documented cases of GGT with concomitant Pulmonary embolism diagnosis that would point to a causal association.

## Introduction

Glomus tumors are rare tumors of mesenchymal origin, usually benign. They are usually found in the distal extremities and rarely seen in the gastrointestinal tract, accounting for <1% of all gastrointestinal soft tissue tumors [1]. Due to their location, glomus tumors are often mistaken for gastrointestinal stromal tumors (GISTs) and other gastric subepithelial lesions, making their diagnosis dependent mostly on pathological and immunohistochemical findings [2].

## Case presentation

Here, we describe a case of a 50-year-old morbidly obese African American male with a past medical history of hypertension, prediabetes, and obesity who presented with a one-month history of shortness of breath and fatigue worsening over the last one week before presenting. He denied fever, chest pain, cough, hemoptysis, or lower extremity edema. He was hemodynamically stable. On presentation, laboratory results were remarkable for severe microcytic anemia with hemoglobin of 2g/dl, mean corpuscular volume (MCV) 55.4fL; the laboratory results were as follows: international normalized ratio (INR) 1.55, Prothrombin time (PT) 18.4s, blood urea nitrogen (BUN) 39mg/dL, Creatine (Cr) 1.74mg/dL. Aspartate aminotransferase/alanine transaminase/alkaline phosphatase (AST/ALT/ALP) 365/621/520 units per liter, T. bilirubin 1.0. Iron studies revealed serum iron <10 and ferritin 38. The bedside guaiac test was positive. He denied any use of aspirin, anticoagulation, melena, hematochezia, or abdominal pain. He has never had a colonoscopy or upper endoscopy in the past. Family history was pertinent for colon cancer in his father at the age of 70.

**Figure 1:**
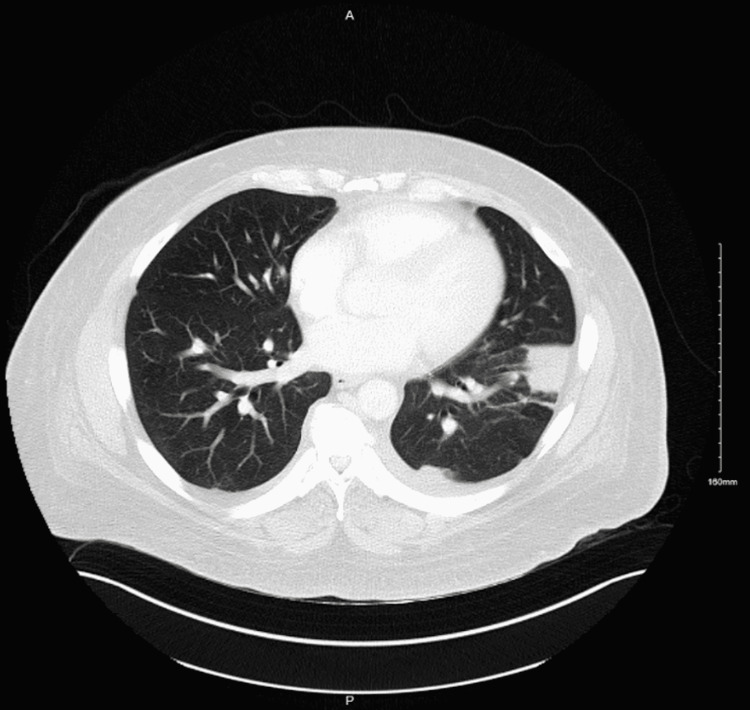
Contrast-enhanced computer tomography depicting left lower lobe pulmonary infarct

**Figure 2:**
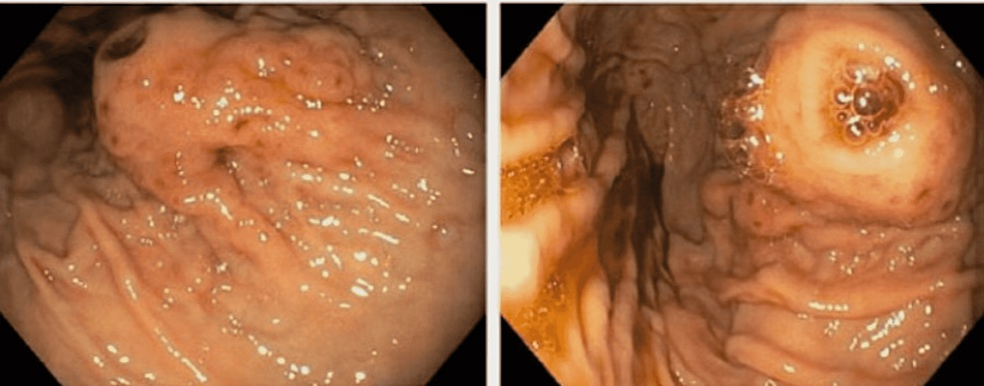
Two cuts from EGD Showing submucosal mass in the body of the stomach EGD: Esophagogastroduodenoscopy

Computed tomography without contrast of the chest and abdomen was given impaired kidney function, which showed a mass at the lung base with no abdominal pathology. He required about 10 units of packed red blood cell transfusion to maintain hemoglobin over 7g/dl in 4 days. Computer tomography with a contrast of chest and abdomen was done following improvement in renal function, which showed a 3.7x2.5cm calcified mass in the stomach, and the left lower lobe mass is previously seen on non-contrast CT was characterized as left lower lobe pulmonary embolism with pulmonary infarction as depicted in figure 1. The patient underwent endoscopy revealing a large submucosal mass measuring 15-20mm in the proximal part of the stomach on the lesser curvature, as seen in figure 2.

**Figure 3:**
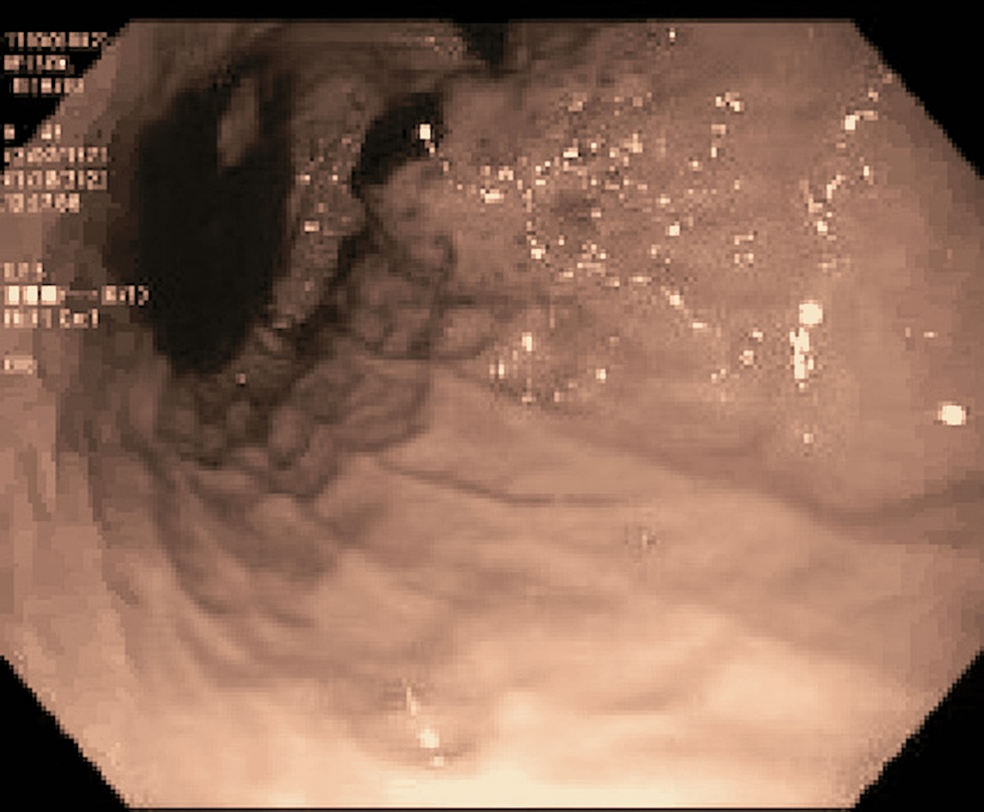
Showing blood oozing from mass

Colonoscopy was also done, showing diverticulosis and non-bleeding hemorrhoids. Initially, the lesion in the stomach was thought to be a gastrointestinal stromal tumor, and biopsies were taken. After excluding an active gastrointestinal bleed, the decision was eventually made to start anticoagulation for pulmonary embolism, but the patient developed hematochezia prompting the treatment to be stopped. He required an additional blood transfusion. Repeat endoscopy showed blood oozing from the mass, as seen in figure 3. 

**Figure 4:**
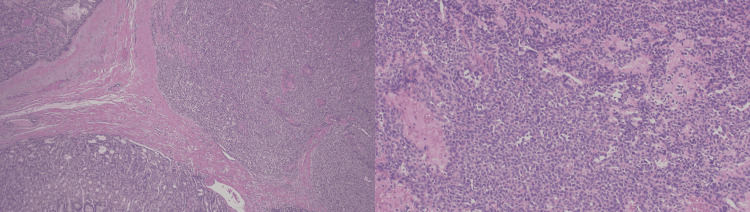
Left. Solid glomus tumor with nodular pattern in the wall of the stomach. The native gastric mucosa is seen in the left lower quadrant (low magnification). Right. Glomus tumor is comprised of uniform cells with round nuclei and eosinophilic cytoplasm (high magnification) with strong expression of smooth muscle actin (not shown).

Preliminary biopsy results showed gastric oxyntic mucosa with polypoid foveolar hyperplasia. Vascular and general surgery were consulted, who agreed to place an IVC filter to address venous thromboembolism to decrease the risk of recurrent PE as anticoagulation was contraindicated due to active GI bleeding. The patient underwent an IVC filter and partial gastrectomy. The patient had an uneventful recovery following surgery, and his hemoglobin improved. He was discharged home with an IVC filter with a close follow-up with gastroenterology, hematology, and oncology as an outpatient. His final pathology result was consistent with the Gastric Glomus tumor. Histological examination of the biopsy specimen is depicted in figure 4. Immunohistochemistry showed that the tumor cells were positive for smooth muscle actin (SMA) and negative for CD117, DOG-1, and CD34 consistent with a diagnosis of gastric glomus tumor.

## Discussion

Glomus tumors are rare entities, and those involving the stomach or gastrointestinal tract are even less commonly reported. It was first reported in a 64 -year-old male Smol'iannikov A in 1928 [3]. The glomus body aids in thermoregulation by promoting blood shunting between the arteries and veins [4]. These soft tissue tumors are mostly found in the upper extremities, with a predilection for the subungual region of the finger [5]. It has a female predominance with a wide age of onset from 19-90 years, more commonly occurring in middle-aged and older adults. The most common site in the stomach is the antrum. 

Gastric glomus tumors can present with a wide range of symptoms. The most common presentations are epigastric discomfort, epigastric pain, and upper gastrointestinal hemorrhage [6]. In a study of 56 patients, the most prevalent clinical symptoms include epigastric pain (61%) and blood in the stool (25%), whereas 14% of patients were asymptomatic [6]; with the asymptomatic patients usually incidentally discovered on endoscopy [6]. Malignant variants are rare; less than 1% of reported glomus tumors [7]. According to classification criteria for malignant glomus tumors, a deep-seated position with a size >2.0 cm, aberrant mitotic figures, or high nuclear grade is proposed as high-risk features [8].

Most gastric tumors are found in the submucosal layer of the stomach and visualized with an EGD. They appear as mucosal elevation and, in some cases, may cause erosion of the mucosal surface resulting in gastrointestinal bleed. Due to their similar appearance to gastrointestinal stromal tumors, they are often misdiagnosed. While traditional endoscopic biopsies can be used to diagnose, they can become challenging due to the intramural placement of these lesions, which makes it difficult to collect an adequate biopsy sample [9]. As a result, endoscopic ultrasonography-guided fine-needle aspiration (EUS-FNA) is being increasingly used for evaluation and sampling [9,10]. 

GGT can be misdiagnosed with other gastric submucosal tumors originating from the mesenchyme, such as GIST, carcinoid tumors, schwannomas [11], hemangiomas of the stomach, and some other rare tumors. Hence, diagnosis is dependent on histopathology and immunohistochemistry findings being the gold standard based on the staining. Immunohistochemical staining is characteristic of the expression of the mesenchymal markers synaptophysin, smooth muscle actin, laminin, and vimentin. Noticeably absent is CD 117 expression, which aids in distinguishing glomus tumor from the more common GIST [12].

Current management involves wedge resection with negative margins. In cases involving tumors at the pylorus, distal gastrectomy with reconstruction is considered to avoid gastric outlet obstruction. Other treatment modalities involve endoscopic methods like endoscopic submucosal dissection [13]. Long-term follow-up is required given the rare malignant potential; however, no consensus guidelines exist. Our patient's endoscopy with a traditional biopsy revealed a gastric glomus tumor; therefore, EUS was not performed. A CT scan revealed a localized tumor with no evidence of metastasis. Complete resection is critical because of the minimal or possible risk of malignant transformation, which was done in our patient. We also hypothesize the possibility of a sub-epithelial lesion such as a gastric glomus tumor as a risk factor for VTE. This patient had no known family history of a hypercoagulable state or any known traditional risk factors.

Malignancy is a known risk factor for the development of pulmonary embolism. With the increasing use of multi-detector-row computed tomography, the incidence of detecting pulmonary embolisms, minimal and sub-segmental ones, has increased many folds. It is risk-stratified based on a scoring system, PESI. It is managed with anticoagulation, but there are instances such as this case when such treatment is contraindicated due to high bleeding risk, and IVC filter placement with reevaluation for removal and starting anticoagulation once appropriate can be considered.

## Conclusions

Gastric glomus tumors are rare, primarily solitary tumors in the submucosal layer with a good prognosis. They may present as an acute gastrointestinal bleed. Given how rare this pathology is, there are no established staging, treatment, or follow-up guidelines. Current treatment practices involve resection with negative margins. There has been a lack of long-term follow-up after surgery; reported surveillance with repeat endoscopy is suggested, given the small but possible risk of recurrence.
